# Factors contributing to human and veterinary medicine shortages in developing countries: perspectives of suppliers and regulators in Namibia

**DOI:** 10.1186/s41182-025-00799-1

**Published:** 2025-10-16

**Authors:** Kavitjiukua Uahupirapi, Saren Shifotoka, Vulika Nangombe

**Affiliations:** 1 School of Pharmacy, University of Namibia, Box 13301, Windhoek, Namibia; 2 Department Pharmacy Practice & Policy, School of Pharmacy, University of Namibia, Box 13301, Windhoek, Namibia

**Keywords:** Medicine shortages, Pharmaceutical supply chain, Public procurement, Regulatory capacity, Veterinary medicines, Namibia

## Abstract

**Background:**

Medicine shortages remain a pervasive global public health challenge, particularly affecting import-dependent countries with limited local manufacturing capacity. Namibia’s reliance on pharmaceutical imports makes it vulnerable to supply chain disruptions across human and veterinary health sectors.

**Aim:**

This study explores availability and factors contributing to medicine shortages in Namibia from the perspectives of regulators and pharmaceutical suppliers.

**Methods:**

An exploratory qualitative study was conducted between August and October 2024. In-depth semi-structured interviews were carried out with 11 key stakeholders in the pharmaceutical sector involved in procurement, distribution, and regulation across public and private sectors, including animal health. Thematic analysis was employed.

**Results:**

Five themes emerged: persistent shortages affecting both sectors with pronounced public sector challenges, particularly for chronic disease treatments (antihypertensives, insulin, anti-tuberculosis medicines, antiretrovirals); general supply chain constraints including limited local manufacturing, small market size, and global active ingredient shortages; public sector barriers including absence of formal procurement contracts and manual systems; regulatory bottlenecks encompassing processing delays and capacity constraints; and veterinary sector vulnerabilities despite economic importance.

**Conclusions:**

Stakeholders identified medicine shortages as resulting from interconnected systemic challenges encompassing procurement, regulatory, infrastructural, and market-based constraints. Key patterns emerged: Namibia’s regional reclassification appears to have increased supply vulnerabilities; absence of formal procurement contracts has led to reliance on emergency mechanisms; and regulatory capacity limitations may compound supply challenges. These interconnected factors suggest the need for coordinated multi-domain interventions, though further research is needed to quantify these relationships. Findings may inform policy considerations for improving medicine security in similar resource-constrained settings.

**Supplementary Information:**

The online version contains supplementary material available at 10.1186/s41182-025-00799-1.

## Background

### Medicine shortages: global context, health impacts, and cross-sector implications

Medicine shortages represent a significant global health challenge. The World Health Organization (WHO) defines a medicine shortage as a situation where the supply of essential pharmaceuticals is insufficient to adequately address public healthcare needs [1]. The issue has intensified globally, affecting both developed and developing nations [2–12]. Shortages in medicine supply disproportionately affect developing countries, particularly in Africa, where healthcare systems face unique challenges [3]. African countries experience higher rates of stockouts compared to developed nations, with studies reporting shortage rates of 25–80% for essential medicines in public healthcare facilities [3–5]. The continent's pharmaceutical landscape is characterised by heavy reliance on imports (up to 90% of medicines), limited local manufacturing capacity, weak regulatory systems, and inadequate supply chain infrastructure [3, 13].

Medicine shortages have profound implications for health outcomes and healthcare delivery. In African settings, shortages have been reported to lead to treatment interruptions, suboptimal clinical outcomes, and increased healthcare costs as patients are forced to seek alternatives in the private sector [6, 7, 12, 14]. Studies from South Africa, Namibia, Kenya, Ghana, and Uganda, among others, demonstrate that medicine shortages result in delayed treatment initiation, reduced treatment adherence, and increased morbidity and mortality rates [12, 14–17]. For chronic conditions such as Human Immunodeficiency Virus (HIV), tuberculosis (TB), and diabetes, which are prevalent in sub-Saharan Africa, treatment interruptions can lead to drug resistance, disease progression, and increased transmission rates [6, 12, 15, 16, 18].

The impact of medicines shortages extends beyond human health to the veterinary sector. In sub-Saharan Africa, the veterinary pharmaceutical market is also largely import-dependent—sourcing primarily from Europe, China, India, and other regions—but remains fragmented due to unharmonised national regulatory frameworks and complex supply chains [19]. Furthermore, major manufacturers have limited presence on the continent, primarily in South Africa and select regional hubs [19]. These shortages disrupt animal health programmes, with adverse implications for food security and economic productivity in agriculturally driven economies [19–22]. The interconnected nature of human and animal health, emphasised through the One Health approach, makes veterinary medicine shortages particularly concerning for the prevention of zoonotic disease [20, 21].

### Southern African context

The Southern African Development Community (SADC) region faces particular vulnerabilities in pharmaceutical security. Countries in this region share similar challenges, including dependence on pharmaceutical imports, with many sourcing primarily from South Africa, which itself experiences significant medicine shortages in its public sector [8–11]. Common regional challenges include procurement inefficiencies, regulatory bottlenecks, inadequate funding, and poor inventory management systems [10, 12, 14, 23]. Studies across sub-Saharan Africa have documented comparable issues with public sector medicine availability, highlighting the regional nature of these challenges [8–10, 24]. These structural vulnerabilities make the region particularly susceptible to global supply disruptions, as evidenced during the COVID-19 pandemic [25–28].

### Namibian context

Namibia, a lower-middle-income country in southern Africa, shares borders with Angola, Botswana, South Africa, and Zambia and has a population of approximately three million people [29, 30]. The national economy is supported by various industries, including beef production, with annual beef exports estimated at 20 million kilograms, primarily destined for the European Union [31, 32]. Namibia operates a two-tier healthcare system comprised of: (1) public health services administered by the Ministry of Health and Social Services (MoHSS) serving the majority of the population, and (2) private healthcare services catering to approximately 15–17% of the population [32]. Pharmaceutical supply to public sector facilities is managed centrally through the Central Medical Store (CMS), which is responsible for the procurement, warehousing, and distribution of medicines and health commodities [32, 33].

Namibia faces considerable public health challenges, including high rates of communicable diseases such as HIV and TB, non-communicable diseases, as well as zoonotic and non-zoonotic animal diseases [31, 32]. The pharmaceutical sector faces several critical challenges, most notably the lack of a sufficiently developed local pharmaceutical manufacturing sector, rendering the country heavily dependent on imports [34, 33]. This dependency creates vulnerability to supply chain disruptions, both regionally and globally, with the majority of pharmaceutical imports coming from neighbouring South Africa, which itself experiences shortages in its public sector [8].

Public procurement in Namibia, including for pharmaceuticals, is governed by the Public Procurement Act, 2015 (Act No. 15 of 2015), which provides a legal framework for procurement and establishes the Central Procurement Board of Namibia (CPBN) to oversee the bidding process for public entities [35]. In 2020, approximately 50% of MoHSS expenditure (about N$4 billion) was allocated to procurement [36]. Despite this substantial expenditure and the existence of a regulatory framework, significant challenges persist in ensuring optimal purchase prices and minimising stockouts [36]. Namibia's total health expenditure accounts for less than 10% of GDP, and this, combined with its relatively small population, limits the country’s purchasing power and competitiveness in the global pharmaceutical market, especially during supply constraints [36–39]. While public sector procurement reforms were introduced in 2015, key issues persist, including the absence of open tendering, frequent stockouts, and reliance on emergency procurement [33]. These constraints directly impact service delivery and undermine progress towards Universal Health Coverage (UHC) [8, 33, 40–42]. Studies recommend the adoption of early warning systems, improved real-time stock monitoring, and more efficient supply chain data use for forecasting and procurement planning to improve medicine availability and resilience [8, 33, 43].

Building on earlier efforts to monitor medicine shortages in Namibia, this study offers a more comprehensive examination of factors contributing to disruptions in the pharmaceutical supply chain [34]. While prior studies in Namibia have documented the occurrence of medicine shortages, few have explored the underlying causes from the perspective of key institutional actors or proposed context-specific solutions [34]. This study addresses that gap by exploring the views of key stakeholders—namely public and private pharmaceutical suppliers and the Namibia Medicines Regulatory Council (NMRC). It seeks to develop a nuanced understanding of the drivers of medicine shortages and identify context-specific potential solutions aimed at strengthening Namibia’s pharmaceutical supply system.

The research was guided by the following specific objectives: firstly, to assess the current state of medicine availability in Namibia from supplier and regulatory perspectives; secondly, to identify the factors that suppliers perceive as contributing to medicine shortages; thirdly, to explore the NMRC’s perspective on the underlying causes of these shortages; and lastly, to examine context-specific potential solutions proposed by suppliers and the NMRC for mitigating medicine shortages in the country.

## Methods

### Study design and theoretical framework

The study adopted an exploratory qualitative design using in-depth, semi-structured interviews. This qualitative approach was selected to gain deep and contextualised insights into stakeholder experiences and perceptions of complex supply chain and regulatory dynamics, dimensions that may not be fully captured through quantitative methods. The research was informed by a health systems framework, which conceptualises medicine availability as an outcome of interactions across three key domains: supply chain resilience, regulatory governance, and multi-stakeholder coordination [44, 45]. This theoretical lens provided a foundation to explore the factors contributing to shortages and identify context-specific potential solutions while remaining open to emergent themes grounded in participants’ narratives [44, 45].

### Study setting and location

The study was conducted in Namibia, a southern African country with a population of approximately 3 million people distributed across 14 regions [29]. Namibia covers 824,292 square kilometres with a population density of 3.7 people per square kilometre, making it one of the least densely populated countries globally [29]. The pharmaceutical sector is largely concentrated in the capital city, Windhoek (Khomas Region), which hosts the regulatory authority, most pharmaceutical importers, wholesalers, and distributors [32]. Given the national scope of medicine distribution and the relatively small number of key stakeholders in the sector, participants were purposively recruited from key regions to ensure broader geographic and institutional representation.

### Study population and sampling

The study population comprised key stakeholders involved in the Namibian pharmaceutical sector, including public and private sector suppliers, veterinary medicine suppliers, and NMRC representatives. Purposive sampling was used to identify participants with direct experience and knowledge of medicine procurement, warehousing, distribution, or regulation in Namibia. Inclusion criteria were: (1) minimum 2 years ’ experience in pharmaceutical supply chain roles or regulation; (2) current involvement in the said roles; and (3) willingness to participate and provide informed consent.

#### Sample size and recruitment process

A total of 19 interview invitations were sent to prospective participants identified via publicly available contact details and professional networks. Invitation emails included an information sheet and consent form, with follow-up reminders sent to non-respondents after 1 week. Of the 19 invitations sent, 15 participants consented to be interviewed, yielding a 78.9% initial consent rate. However, interviews were ultimately conducted with 11 participants, as four who had initially consented were later unavailable during the data collection period, resulting in a final participation rate of 73.3% among those who consented.

While sample sizes in qualitative research vary, with 20 often referenced, the principle of data saturation guided our approach [46]. As it is accepted that sample size can be guided by thematic saturation, study population characteristics, and the complexity of the phenomenon being explored [46]. Given the relatively small size of Namibia’s pharmaceutical sector, with an estimated 15 to 20 major suppliers and a limited number of regulatory personnel, the available pool of eligible participants was inherently limited [47]. Data saturation was reached after eight interviews, with no new themes emerging. Three additional interviews were conducted to ensure comprehensive representation of all key stakeholder groups and validate emerging themes.

### Geographical distribution of participants

Participants were recruited from multiple sites across Namibia to ensure regional and sectoral representation, as summarised in Table 1. Nine participants (*n*=9, 81.8%) were based in Windhoek (Khomas Region), reflecting the concentration of pharmaceutical supply, regulatory oversight, and veterinary wholesaling activities. Two participants were recruited from the northern Oshana region: one from Ondangwa and the other from Ongwediva, representing private sector suppliers involved in regional pharmaceutical distribution. This distribution enabled the inclusion of both central and peripheral stakeholder perspectives, while acknowledging that pharmaceutical regulatory and supply chain decision-making in Namibia is largely centralised in Windhoek due to its regulatory, infrastructural, and commercial significance.

**Table 1:** Participant characteristics by sector and location

Stakeholder	Health sector	Location	Number
Regulators	–	Windhoek^a^	2
Suppliers	Private, human	Windhoek^a^	4
		Ondangwa^b^	1
		Ongwediva^b^	1
	Private, veterinary	Windhoek^a^	1
	Public, human	Windhoek^a^	2
Total			11

^a^Khomas region

^b^Oshana region

### Data collection

Data collection was conducted between August and October 2024 using semi-structured interviews. Face-to-face interviews were conducted at participants’ workplaces, while online interviews were done via video conferencing (Google Meet^®^) due to logistical constraints or participant preferences.

#### Interview guide development and procedures

The interview guides (Appendices B and C) were developed based on existing literature and were pilot tested with two stakeholders who were not included in the final sample [48]. Separate interview guides were developed for different stakeholder groups (regulators and suppliers) but covered common domains including current state of medicine availability, perceived drivers of shortages, regulatory challenges, and proposed interventions.

All interviews were conducted following the interview guides in English by a female final-year Bachelor of Pharmacy student, who received in-house orientation and was guided by supervisors. The interviewer had no prior relationship with the participants; her student status and the academic purpose of the study were explained to all participants. Interviews lasted 45–90 min (average 42 min) and were audio-recorded with participant consent. No repeat interviews were conducted. Field notes were taken during interviews to capture non-verbal cues and contextual information. All participants verified their interview transcripts for accuracy as part of the member checking process.

### Data analysis

All interview audio recordings were transcribed verbatim using Microsoft Word^®^’s online transcription tool and verified for accuracy by the research team. The transcripts were analysed using an inductive thematic analysis approach, following the six-phase process outlined by Braun and Clarke (2006): (1) familiarisation with data; (2) generating initial codes; (3) searching for themes; (4) reviewing themes; (5) defining and naming themes; and (6) producing the report [46]. This approach enabled the identification of patterns and insights grounded in participants’ lived experiences regarding medicine shortages and potential solutions, without imposing predefined categories. However, coding and interpretation were loosely informed by both the study objectives and the health systems theoretical framework, which acted as a sensitising lens during theme refinement to ensure comprehensive coverage of supply chain, regulatory, and coordination factors.

#### Coding process and theme development

Two researchers independently coded the transcripts to ensure consistency and reliability. Initial coding was performed line-by-line, with codes subsequently grouped into potential themes through iterative analysis that considered factors contributing to shortages and proposed solutions from both supplier and regulatory perspectives. Discrepancies between coders were resolved through discussion and, when necessary, consultation with a third researcher.

Themes were developed based on frequency of occurrence, relevance to the study objectives, and conceptual coherence within the health systems framework’s three domains. The final thematic framework was mapped against the study objectives to ensure comprehensive analytical coverage of current medicine availability, contributing factors, regulatory perspectives, and potential solutions. Although the theoretical framework informed the thematic structure at a broader level, the coding process remained fundamentally data-driven to capture context-specific dynamics relevant to Namibia’s pharmaceutical landscape.

#### Data management

Data were managed and organised manually using Microsoft Word with systematic file naming conventions and version control. A detailed audit trail was maintained throughout the analysis process. Verbatim participant quotations are presented in italics and enclosed in quotation marks. Minor grammatical edits are shown in parentheses to improve readability; ellipses indicate omitted content. Where appropriate, participant roles and affiliations are included to contextualise perspectives while maintaining anonymity. All data and analytic records were securely stored in password-protected folders accessible only to the research team.

### Ethical considerations

Ethical approval was obtained from the University of Namibia School of Pharmacy and the Ministry of Health and Social Services Research Ethics Committee (Reference number: 22/3/1/2). All participants provided written informed consent before interviews. Confidentiality was maintained through anonymisation of transcripts and secure data storage. Participants were informed of their right to withdraw at any time without consequences.

### Study limitations

This study has limitations that should be considered when interpreting the findings. The 73% response rate may limit representativeness, as non-participants could have offered differing views. While thematic saturation was achieved, it may not fully capture the diversity of perspectives within Namibia’s pharmaceutical sector, particularly those of smaller private suppliers, regional public depots, and public veterinary suppliers. Geographic representation was limited to participants with accessible contact information who either resided in Windhoek or had reliable video conferencing access, thereby excluding some remote areas despite these being serviced by CMS or urban-based suppliers. Some interviews were conducted online, which may have affected rapport and depth of responses. The cross-sectional design captures perspectives at a single time point and does not account for seasonal variations in availability or any recent policy or other changes that could influence stakeholder experiences. The study focused solely on supply-side stakeholders, excluding demand-side viewpoints from healthcare providers, patients, and end-users. The veterinary sector was represented by only one participant, limiting the comprehensiveness of insights into animal health medicine challenges despite this sector’s economic importance. Reliance on self-reported perceptions without triangulation with objective medicine shortage data may introduce reporting bias.

## Results

### Participant characteristics

A total of 11 interviews were conducted with key stakeholders in Namibia’s pharmaceutical sector. The sample comprised representatives from four key stakeholder groups: Namibia Medicines Regulatory Council (NMRC) officials (*n* = 2), Central Medical Stores (CMS) representatives (*n* = 2), private pharmaceutical suppliers (*n* = 6), and veterinary medicine suppliers (*n* = 1). The sample included 6 females and 5 males, with participants having a median of 6 years of experience in the pharmaceutical sector (range: 2–25 years). Most participants (*n* = 8, 73%) were pharmacists holding managerial or senior technical positions within their respective organisations. The remaining participants (*n* = 3) held operational roles in pharmaceutical procurement, distribution, or regulatory affairs. All participants met the inclusion criteria of having a minimum of 2 years’ experience in pharmaceutical supply chain roles or regulation and current involvement in their respective roles. All participants provided informed consent and were assured of confidentiality through the use of anonymous participant codes (NMRC01-02, CMS01-02, S01-S07) throughout data analysis and reporting.

### Thematic findings

Thematic analysis process yielded five key themes that collectively address all four study objectives. Theme 1 directly addresses the current state of medicine availability in Namibia (Objective 1), Themes 2–4 explore contributing factors from supplier and regulatory perspectives (Objectives 2–3), and Theme 5 examines proposed solutions from all stakeholder groups (Objective 4).

### Theme I: current state of medicine availability in Namibia

#### Sectoral disparities in medicine access

All participants acknowledged notable disparities in medicine availability between sectors, with the public sector described as experiencing more severe shortages than the private sector. This perceived disparity suggests fundamental differences in procurement systems, financial resources, and supply chain management capabilities between sectors. An NMRC official characterised the overall situation as follows: “*The availability of medicine is very, very low. Currently, we are experiencing shortages of medicines in Namibia.”* A CMS representative corroborated this assessment, describing the public sector situation as *“significantly challenging.”* (CM01).

In contrast, private sector suppliers reported more favourable availability, though with emerging systemic vulnerabilities. One private supplier stated: “ *from [a] private sector point of view, I do not think lack or shortage of medicine is a problem*” (S01), while another noted: “… *medicines are always available but in the public sector it [is] quite challenging and medicines are out of stock*” (S07). However, some private sector participants expressed growing concerns about future supply security: *“In the private sector, the availability of medicine isn't a major problem, but it is becoming concerning due to factors like political turmoil and load shedding in South Africa, where most medicines are sourced. These issues lead to frequent stock shortages. In the state sector, the situation is much worse.”* (S03).

This emerging vulnerability suggests a potential shift from historical public–private disparities toward system-wide risks, as both sectors depend heavily on South African supply chains that are increasingly subject to external disruptions.

#### Medicine categories most affected by shortages

Participants identified specific therapeutic categories consistently affected by shortages across sectors. Table 2 presents the medicines most frequently reported as out of stock or in short supply by sector. Analysis of shortage patterns revealed both common vulnerabilities across sectors and sector-specific challenges. Chronic disease medications, particularly antihypertensives, antiretrovirals (ARVs), insulin, and anti-tuberculosis medicines, were consistently reported as problematic across both public and private sectors, reflecting both high demand and complex global supply chain dependencies.

**Table 2:** Medicines frequently affected by shortages in Namibia

Medicine category	Private sector	Public sector	Regulator observations	Animal health sector
Antihypertensives	✓	✓	✓	–
Antiretrovirals (ARVs)	✓	✓	–	–
Insulin/diabetes medicines	✓	✓	–	–
Anti-TB medicines	✓	✓	–	–
Oncology medicines	✓	–	–	–
Antipsychotics	✓	–	–	–
Antibiotics	–	✓	–	–
Antihistamines	–	✓	–	–
Antidepressants	–	✓	–	–
Anti-cholesterol medicines	–	–	✓	–
Antimalarials	–	–	✓	
Vaccines	–	–	–	✓

The veterinary sector presented a distinct pattern, with shortages described as selective rather than systematic. The veterinary supplier explained: "*Not with all medicine, in the animal health the availability is not consistent*" (S04). Vaccines were particularly affected due to manufacturer prioritisation decisions during global health crises, highlighting the sector’s vulnerability during emergencies when human health takes precedence.

#### Impact of COVID-19 on medicine availability

The COVID-19 pandemic emerged as a critical inflection point that exacerbated existing vulnerabilities and created new supply chain challenges. Participants consistently described how the pandemic disrupted global pharmaceutical supply chains through multiple mechanisms, including active pharmaceutical ingredient (API) shortages, increased transportation costs, and manufacturing prioritisation shifts. One supplier detailed these interconnected impacts: *“There are more random [medicine] shortages due to API shortages from the manufacturer. Cargo has also increased prices of products from India and China”* (S06). This observation illustrates how the pandemic created cascading effects throughout the global pharmaceutical supply chain, with raw material shortages combining with logistical disruptions to compound supply challenges.

The veterinary sector was described to experience severe impacts, as one participant explained: “*…COVID[-19] is also affecting our vaccines to this day, maybe [because] some manufacturers were driven towards saving humans and forget about animals (sic)”* (S04). This prioritisation pattern reflects rational resource allocation during a global health emergency but highlights the vulnerability of non-human health sectors during crises.

#### International comparisons and patient impact

Participants provided nuanced perspectives on Namibia's relative position regarding medicine availability globally and regionally. While acknowledging that medicine shortages are a global phenomenon, several participants believed that Namibia lags behind international standards. However, regional comparisons suggested a more complex picture, with some respondents noting that Namibia's situation is relatively better than that of some neighbouring countries such as Angola, Zambia, and Zimbabwe, but comparable to Botswana and South Africa.

All participants acknowledged significant negative impacts of medicine shortage on patients, including treatment interruptions that can lead to disease progression, increased transmission of communicable disease, elevated morbidity, and mortality. Private sector patients sometimes accessed alternatives, though these were often more costly or less clinically effective, creating additional barriers to optimal care and potentially exacerbating health inequities.

### Theme II: pharmaceutical supply chain challenges across sectors

#### Market size limitations and procurement delays

Namibia’s relatively small population (approximately 3 million) emerged as a fundamental structural challenge that affects all aspects of pharmaceutical supply. Multiple participants identified how this market size limitation makes it difficult to secure medicines, as international manufacturers prioritise larger more profitable markets when allocating limited supplies. One supplier explained the practical implications: “*Long lead times for medicines to come to Namibia and Namibia being a small market and it does not meet the minimum order quantity.”* (S07). This challenge is compounded by extended procurement lead times, as noted by an NMRC representative: “*Procurements lead times of manufacturers taking long to provide the medications (sic)”* (NMRC02). These interconnected challenges create a cycle where small market size leads to longer procurement times, which in turn make supply planning more difficult and increase the risk of stockouts during the extended procurement periods.

#### Dependence on imports and manufacturing constraints

The limited local pharmaceutical manufacturing capacity emerged as a critical vulnerability that makes Namibia dependent on international suppliers and susceptible to external disruptions. This dependency was characterised by participants as a strategic weakness that affects both supply security and negotiating power. One participant highlighted the prioritisation challenges this creates: “*The lack of a local manufacturing [capacity] is a major issue. While [Namibia-based pharmaceutical suppliers] rely on imports, being small businesses means they are often not prioritised in receiving supplies.”* (S04). Another noted broader market withdrawal trends: *“A lot of companies [are] just not willing or not being able to continue with their business in Namibia, a lot are pulling their products from the market.”* (S03).

A particularly significant development was Namibia’s reclassification in global supply chains. As one supplier explained: *“Multinational companies have withdrawn from the Namibian market due [to] small population size, last year [2023] and this year some of the suppliers, the manufacturers no longer regard us [Namibia] as South African import country, they regard us sub-Saharan export country”* (S05)*.* This reclassification represents a fundamental shift in supply chain positioning that potentially increases costs, extends lead times, and reduces supply security by moving Namibia from an integrated regional market to a peripheral export destination.

#### Global active pharmaceutical ingredient shortages

Participants consistently identified global API shortages as a contributing factor to local medicine shortages, reflecting Namibia’s integration into global pharmaceutical supply chains and vulnerability to upstream disruptions. As one participant noted: *"There're longer lead times and these API constraints"* (CM01). This challenge highlights how local medicine availability is increasingly dependent on global pharmaceutical production systems, making even well-functioning local supply chains vulnerable to disruptions originating in major API production centres, particularly in Asia.

### Theme III: public sector-specific structural and operational barriers

#### Absence of formal procurement contracts

A critical structural barrier emerged in the form of CMS operating without formal pharmaceutical procurement contracts due to ongoing legal disputes between the Central Procurement Board of Namibia (CPBN) and previously contracted suppliers. This situation creates fundamental uncertainty in the public pharmaceutical supply chain. A CMS participant explained the situation: *“The government [pharmaceutical acquisition] at the moment is running on emergency [procurement]. Instead of contracts, and this is because of the CPBN contracting which is still going on in court and is not finalised.”* (CMS02). This emergency procurement approach creates multiple challenges, including lack of supply predictability, potential cost increases, limited supplier accountability, and administrative inefficiencies. A private sector participant provided additional context on systemic procurement challenges, citing loopholes and "tenderpreneurs" (businesspeople exploiting government tenders) as key issues [49]: *“An inadequate procurement system that the government uses. There are many loopholes, tenderpreneurs that partake in the procurement process for the government especially*,* and [a] prime example is now again like the tender that's not being adjudicated (as) one company can cause the whole country not to have medication (sic)’’.* (S03).

#### Legislative and procedural limitations

The Public Procurement Act, 2015 (Act. No.15 of 2015) was identified as creating structural barriers that impede efficient pharmaceutical procurement. Key limitations include the N$25 million threshold requiring CPBN involvement, which removes procurement autonomy from fundamental entities like CMS, and a price-focused procurement approach that may not adequately consider supply reliability, quality, or service factors. One CMS participant contrasted current limitations with previous operational autonomy: *“Before the Procurement Act, we had an exemption. We could run our own tender. Do everything and report back to tender [CPBN]…”* (CMS02). This change represents a shift from specialised, sector-specific procurement toward centralised, generalised procurement processes that may not be optimally suited for pharmaceutical supply chain management.

#### Manual procurement systems and infrastructure constraints

The manual procurement systems within CMS were identified as an efficiency barrier that slows processes and increases error risks. One CMS participant highlighted infrastructure needs: *“improvements into our procurement systems because our things [sic] are quite manual. If things were automated, it would be faster and better for things to be to move smoothly”.* (CMS01). These manual systems create bottlenecks in procurement processes, limit real-time visibility into inventory levels and procurement status, and increase administrative burden. Financial constraints from inadequate budget allocations and poor inventory management practices compound these challenges, as noted by an NMRC representative: *“supply chain disruptions and procurement. Budgetary constraints and then also poor stock management*” (NMRC02)***.***

### Theme IV: regulatory system capacity constraints and process inefficiencies

#### Infrastructure and human resource limitations

NMRC representatives acknowledged significant capacity constraints that affect their ability to perform core regulatory functions effectively. These constraints encompass both human resources and technological infrastructure, creating systemic bottlenecks in medicine registration and monitoring processes. One NMRC representative summarised the scope of challenges: *“We are understaffed, infrastructure challenges, we do everything manually, we don't have tracking systems ”* (NMRC02). The absence of medicine shortage monitoring systems was specifically mentioned as a critical gap: *“We do not have a run system at this time point and it makes tracking and managing of medicine applications difficult.”* (NMRC02). These capacity constraints create cascading effects throughout the pharmaceutical system, as regulatory delays can prevent medicines from entering the market and limit the ability to respond rapidly to shortage situations through emergency approvals or expedited registrations.

#### Registration and approval process delays

Several aspects of Namibia’s regulatory framework were identified as contributing to delays in medicine availability. The Medicines and Related Substances Control Act, 2003 (Act No. 13 of 2003) was described as containing outdated provisions that constrain regulatory efficiency: *" there are things in the Act that's not practical anymore which constrain us to some extent. Our Acts are actually very old and there are loopholes”* (S05).

Namibia’s classification as a Zone 4b stability country creates additional barriers for pharmaceutical companies seeking market entry. This classification requires companies to conduct additional stability studies specific to Namibian conditions compared to other countries in the region, increasing the time and cost of market entry. One participant explained the practical implications: *“…This requires companies to conduct additional stability studies, rather than just one study for the entire Southern Africa region. As a result, companies must invest more time and money into research, leading many to decide it's not worth the effort for the population size, often opting out of providing their products”* (S03).

Additional delays were reported in medicine registration periods, Section 27 compassionate clearance application approvals, and registration application backlogs. One supplier noted: *“There is such a delay in registering with the new medicines… if we want to import those medicines on a non-registered compassionate clearance basis getting permits takes too long ” * (S05).

### Theme V: proposed multi-faceted solutions for addressing medicine shortages

#### Strengthening regulatory capacity and systems

Participants proposed measures to enhance NMRC’s regulatory capacity, recognising that regulatory efficiency is fundamental to medicine availability. Recommendations focused on three key areas: human resources, infrastructure, and process optimisation. An NMRC representative outlined specific capacity-building needs: *“We [NMRC] should place inspectors at different border posts and can also employ competent specialised assessors to push (sic) so that this medication gets registered and reduce the process of [allowing] unregistered medication. Upgrade our [quality surveillance] lab[oratory]”* (NMRC01). The implementation of real-time national databases for tracking medicine availability was strongly recommended across stakeholder groups, along with expedited approval processes for addressing critical shortages. These technological solutions were seen as essential for transitioning from reactive to proactive shortage management.

#### Public procurement system reform

CMS participants advocated for fundamental reforms to public procurement systems, emphasising the need for digitalisation and restoration of specialised procurement autonomy. These recommendations reflect recognition that pharmaceutical procurement requires specialised expertise and processes different from general public sector procurement. One CMS participant articulated the desired approach: “*We could run our own tender. Do everything and report back to tender [CPBN] so we had a contract running on the system… availability as long we have a contract, and the supplier knows that I have this item.”* (CMS01). Enhanced coordination between CMS and CPBN was proposed to address procurement delays and improve contract management, suggesting a hybrid approach that maintains oversight while enabling specialised procurement expertise.

#### Enhance stakeholder collaboration and coordination

Participants emphasised the need for improved collaboration among key institutions, recognising that medicine shortages result from systemic challenges that require coordinated responses. This theme reflects an understanding that individual institutional improvements, while necessary, are insufficient without improved inter-institutional coordination. A NMRC representative summarised this need: *“NMRC [should] collaborate with partners, stakeholders, and the MoHSS to address medicine shortages. A coordinated effort is needed to effectively tackle procurement issues and optimise preparation for emergencies”* (NMRC02). Specific coordination improvements were proposed for CPBN to address public procurement delays: *“CPBN should be involved in addressing supply issues, as they play a crucial role in ensuring a consistent supply through long-term contracts…Collaborating to identify and resolve these issues could improve the situation for everyone involved”* (CMS01).

#### Technology-enabled solutions and private sector learning

Private sector participants highlighted successful strategies including accurate demand forecasting, real-time stock monitoring, and diversified sourcing as potential models for public sector adoption. These recommendations reflect recognition that private sector supply chain management practices offer valuable lessons for public sector improvement. Technology-enabled inventory management was specifically mentioned as a mechanism to prevent stockouts and improve supply chain responsiveness. The involvement of key government bodies, including the Ministry of Health and Social Services and the Ministry of Finance, was proposed to enhance policy alignment and ensure adequate resource allocation for public pharmaceutical procurement.

## Discussion

Medicine shortages remain a pervasive global public health challenge, influenced by a complex array of factors across contexts and health systems [2, 50]. These factors encompass supply chain gaps, regulatory hurdles, demand forecasting challenges, and broader systemic issues [3, 10]. In developing countries, particularly across Africa, these structural challenges are often exacerbated by high import dependency, limited local manufacturing, weak regulatory systems, and inadequate infrastructure [3, 10, 28]. This study is the first to explore medicine shortages in Namibia from the perspectives of national regulators and pharmaceutical suppliers, spanning both human and veterinary sectors. It builds on Rennie et al.’s earlier findings on shortages from demand-level staff in community, hospital, and wholesale pharmacy settings to provide a comprehensive understanding of these complex factors in a specific African context [34].

### Current state of medicine availability in Namibia (Theme I)

Participants confirmed that persistent shortages affect both human and veterinary sectors, with pronounced challenges in the public sectors. Shortages of essential chronic disease treatments—particularly antihypertensives, insulin, anti-tuberculosis medicines, and antiretrovirals—reflect findings in other LMICs [50–53]. In Namibia, these disruptions were attributed to procurement insufficiencies, systemic supply chain constraints, and over-reliance on imported medicines, findings corroborated by the Ministry of Finance Procurement Policy Unit [33]. Price inflation and product withdrawal were also noted, including the exit of a major insulin supplier, necessitating suppliers to seek alternatives outside Africa. Nakambale et al. found that 77% of products in a basket of vaccines, ARVs, and TB medicines sourced from Namibian suppliers were more expensive than those from international suppliers. Over half of all products which had both local and international supplier prices were higher than the international reference median buyer prices [54]. A 2009 Root Cause Analysis reported marked price differentials of 62–78% when compared to international and regional pooled procurement mechanisms [33]. These disruptions impact continuity of care and treatment outcomes. This is consistent with evidence from South Africa, Rwanda and other LMICs showing similar public sector stockouts [33, 50–54]. Studies found that public sector stockouts drive patients to seek medicines from the more costly private sector [9].

Namibia is a major exporter of live animals and beef, with the veterinary sector contributing approximately NAD 266.1 million to its economy in 2022 [55]. Despite its economic importance, the sector experiences erratic availability of veterinary medicines, cited to be largely due to high dependency on imported products and the disproportionate prioritisation of human over animal health. This imbalance may compromise food security, exacerbate antimicrobial resistance, and reduce agricultural productivity [56].

### Multifaceted contributing factors to medicine shortages (Themes II–IV)

#### General pharmaceutical supply chain challenges (Theme II)

Namibia’s limited local manufacturing capacity, small market size emerged as major constraints, rendering it reliant on imported products yet limited in its ability to meet minimum order quantities and incentivising supplier product withdrawals. These challenges are compounded by long lead times and global shortages of APIs [33]. These findings resemble those from Finland and Israel, where small markets faced similar vulnerabilities [57, 58]. Limited domestic manufacturing capacity remains a key constraint, with Namibia’s sole manufacturer unable to meet national demand. This is not unique to Namibia; Mozambique also has one operational local manufacturer [59]. Across Africa, only 3% of global medicines are produced, with up to 90% imported [3]. API shortages remain a complex global issue, stemming from the limited number of suppliers, primarily located in India and China [60]. These findings confirm the structural nature of the medicine supply challenge in small and import-dependent markets, exposing countries like Namibia to external shocks and supply disruptions.

#### Public sector structural and operational barriers (Theme III)

In the public sector, the absence of formal procurement contracts, due to ongoing litigation between suppliers and the Central Procurement Board, has forced reliance on emergency “buyouts”. Such mechanisms limit supplier accountability and cost efficiency. Participants also cited the manual nature of procurement systems, lack of digital inventory tracking, and weak coordination with CPBN and other actors as barriers to efficiency [39, 61]. Similar findings have been reported in Nigeria and other LMICs, where centralised procurement is undermined by delayed payments, weak oversight, and limited procurement autonomy [8, 62–64]. Although Namibia’s 2021 Turnaround Strategy proposed critical reforms to modernise the public procurement process, such as e-procurement, publishing of bids and detailed tender results, and threshold revision based on actual pharmaceutical procurement needs, effective implementation is lacking [33]. In many LMICs, public procurement systems often lack accountability, with limited open competition, inadequate transparency, and insufficient application of market-based practices [65]. Studies have also reported limited staff understanding of procurement processes and limited treasury funding as critical barriers [66, 67]. Notably, even in structured systems such as China's centralised medicine procurement model, implementation gaps, e.g., inaccurate demand forecasting and inefficient funding management, can hinder effectiveness [68]. While centralised procurement approaches have been found to reduce costs and stockouts, their success hinges on effective implementation and oversight [69].

#### Regulatory system bottlenecks and capacity constraints (Theme IV)

Regulatory bottlenecks compound these supply chain issues. Participants pointed to medicines regulatory system shortcomings, including delays in processing exemption and compassionate use applications, infrastructure gaps, outdated guidelines, and human resource constraints. Regulatory fragmentation and weak inter-agency collaboration were also flagged. Similar challenges have been reported in Nigeria, where weak regulatory enforcement affects medicine quality and availability, reinforcing the systemic nature of regulatory capacity limitations across African contexts [55]. While Namibia has piloted medicine availability monitoring tools, they remain underutilised [34]. In contrast, Turkey’s successful pharmaceutical track-and-trace system demonstrates how integrated digital infrastructure and coordinated oversight can reduce shortages and improve supply security [55].

#### Proposed multi-faceted solutions for addressing medicine shortages (Theme V)

The proposed solutions from participants align closely with international best practices and existing policy frameworks [3, 8, 62, 70]. Priorities include strengthening NMRC’s human and technical capacity, revising legislative provisions, digitising procurement processes, and establishing a real-time national shortage monitoring system. Participants further recommended long-term supplier contracts, budget ring-fencing for essential medicines, fostering regional pooled procurement via SADC and other mechanisms, and investing in local production [33, 55, 56, 70].

Of note the veterinary sector findings, often overlooked, highlight the interdependence between human and animal health. Failure to safeguard veterinary medicine supply risks zoonotic disease spread and food insecurity, particularly in livestock-based economies such as Namibia [55, 56]. This underscores the relevance of One Health approaches in addressing cross-sectoral medicine security.

The limitations of this study, as outlined in the “Methods” section under “Study limitations”, should be considered when interpreting these findings.

## Conclusions

This study provides a stakeholder-informed analysis of medicine shortages in Namibia across human and veterinary sectors. Our findings indicate that these shortages reflect multiple interconnected systemic challenges, including procurement, regulatory, infrastructural, and market-based constraints. The analysis identifies several concerning patterns. Namibia’s reclassification in regional supply chains appears to have contributed to longer lead times and reduced supplier commitment, though the full extent of this impact requires further investigation. The absence of formal procurement contracts has led to increased reliance on emergency procurement, which may undermine supply predictability and cost-effectiveness. Additionally, misalignment between regulatory capacity and system demands appears to create bottlenecks that compound rather than resolve supply chain challenges.

The public sector experiences substantial challenges through structural inefficiencies and procurement difficulties, while the private sector, despite greater stability, faces vulnerabilities from external supply chain disruptions. In the veterinary sector, prioritisation of human medicines may create risks for animal health and food security, highlighting potential interdependencies that warrant further examination.

Several limitations should be acknowledged. The study's reliance on stakeholder perceptions may introduce bias, and the cross-sectional design limits causal inferences about identified relationships. The sample, while diverse, may not fully represent all relevant stakeholder perspectives across Namibia's health system. Additionally, the study did not quantitatively measure shortage frequencies or economic impacts, limiting the ability to assess the magnitude of identified problems. The veterinary sector analysis was more limited in scope, and findings may not be generalisable to other LMICs without similar contextual factors.

The interconnected nature of the challenges identified suggests that addressing medicine shortages in Namibia may require coordinated interventions across multiple domains. The alignment between stakeholder-proposed solutions and established international practices provides a potential foundation for evidence-based interventions, though adaptation to local contexts would be essential. Further research is needed to quantify the impacts of identified issues and evaluate the effectiveness of proposed solutions.

The study contributes to understanding medicine security challenges in resource-limited settings and highlights the importance of considering both human and veterinary medicine systems in shortage analyses. However, additional research using quantitative methods and longitudinal designs would strengthen the evidence base for policy interventions.

## Recommendations

To address these challenges and ensure consistent medicine availability, we recommend a coordinated approach focusing on six priority areas. First, regulatory capacity must be strengthened through expanded NMRC staffing and infrastructure, streamlined marketing authorisation processes, and revised legislative frameworks. Second, a centralised, real-time shortage monitoring system should be established and coordinated by MoHSS and NMRC for proactive shortage management. Third, revise the Public Procurement Act, digitise CMS operations, and finalise ongoing litigation to restore formal contracting mechanisms [33]. Fourth, promote domestic pharmaceutical production through policy incentives, investment support, and strategic public–private partnerships to reduce import dependence [71, 72]. Fifth, enhanced stakeholder collaboration must be fostered among agencies, suppliers, and regional partners, with attention to SADC pooled procurement mechanisms. Finally, implement longitudinal research to track shortage patterns and evaluate interventions, providing evidence for ongoing policy development. Effective implementation of these interconnected actions will strengthen medicine security across both human and veterinary health systems in Namibia.

## Supplementary Information

Supplementary material 1.

## Data Availability

The datasets used and analysed during the study are available from the corresponding author on reasonable request.

## Abbreviations

API: Active pharmaceutical ingredient
ARVs: Antiretrovirals
CMS: Central Medical Stores
CPBN: Central Procurement Board of Namibia
HIV: Human Immunodeficiency Virus
LMICs: Low- and middle-income countries
MoF: Ministry of Finance
MoHSS: Ministry of Health and Social Services
NMRC: Namibia Medicines Regulatory Council
SADC: Southern African Development Community
WHO: World Health Organization
