# Supplementary material for: Factors contributing to human and veterinary medicine shortages in developing countries: perspectives of suppliers and regulators in Namibia
Source: Trop Med Health. 2025 Oct 16;53:139. doi: 10.1186/s41182-025-00799-1 (PMC12529813; doi:10.1186/s41182-025-00799-1)
Supplement: Supplementary file 1 — Supplementary material 1. [file 41182_2025_799_MOESM1_ESM.docx]

**APPENDICES:**

**APPENDIX A- ETHICS APPROVAL LETTER**


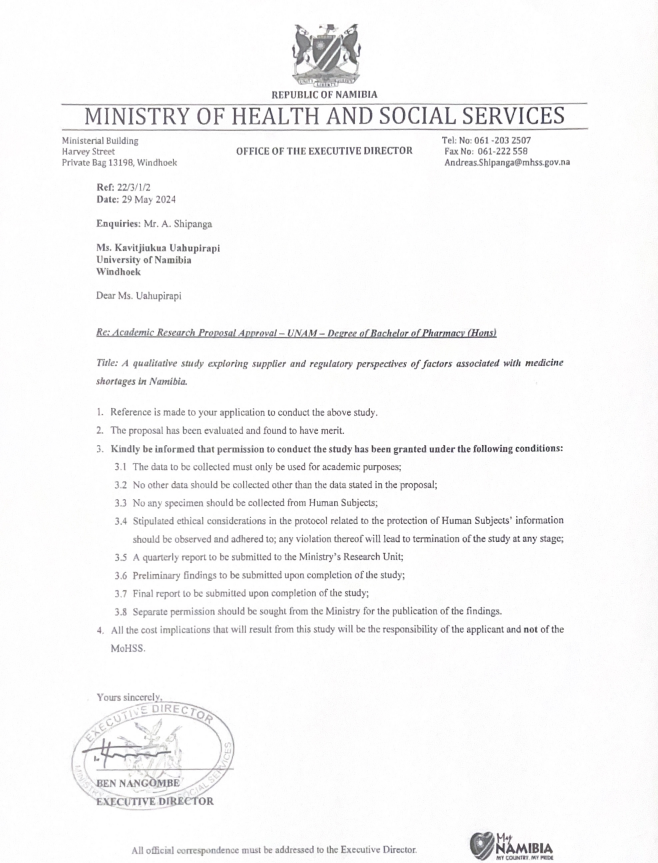

**APPENDIX B – INTERVIEW GUIDE – NMRC**

**PART I: CONSENT SCRIPT:**

Introduction: Welcome and thank you for agreeing to be interviewed. As previously mentioned, my name is xxxx, and I am a fourth-year Bachelor of Pharmacy student at the University of Namibia.

**Interview conduct and ethics:** As communicated during the recruitment process, the interview should take between 30-40 minutes. All information obtained during the interview will be treated with strict confidentiality. Your identity will be kept anonymous, and any personal information shared during the interview will be used solely for research purposes. The interview is being recorded to help ensure complete and accurate documentation of your responses, and it will be destroyed after transcribing it and only the researcher and authorised personnel involved in this study will have access to the data.

**Risks and benefits:**

1. There are no risks involved. However, if some questions evoke emotional responses or discomfort at any point during the interview and you feel uncomfortable or distressed, you have the right to decline to answer any question or terminate the interview.
2. There are no direct benefits to you for participating in this study. However, your contribution will assist in advancing knowledge around medicine shortages in Namibia and may benefit future health service provision and research endeavours.

**Voluntary participation:**

Your participation in this study is entirely voluntary. You have the right to refuse participation or withdraw from the study at any time without providing a reason.
Do you have any questions?
Do you give your informed consent to be interviewed? YES/NO
-----------------

**GENERAL QUESTIONS**
**Participant’s role and experience:**

|  | **Department** | **Position** | **Years of experience in regulatory role** |
| --- | --- | --- | --- |
| **1.** |  |  |  |
| **2.** |  |  |  |
| **3.** |  |  |  |
| **4.** |  |  |  |

# **2.** **MEDICINES AVAILABILITY IN NAMIBIA**

2.1 How would you describe the current state of medicines availability in Namibia?

1. *Please provide examples to illustrate this.*

2.2 How does this situation compare globally?

2.3 How does current medicine availability in Namibia compare to the past 5 years?

2.4 Which medicines or classes are frequently out of stock or in short supply in Namibia?

1. *What are the reasons for these stockouts/ shortages?*

2.5 Which medicines or classes are less affected by supply shortages?

2.6 Do medicine shortages affect the cost of medicines in Namibia?

1. *If so, how?*

2.7 How do medicine shortages affect patients in the public sector?

2.8 How do medicine shortages affect patients in the private sector?

# **3.** **FACTORS ASSOCIATED WITH MEDICINE SHORTAGES**

3.1 In your opinion, what are the top three causes of medicine shortages in Namibia?

1. *Are some of these most problematic?*
2. *Which ones and why?*

3.2 Are the causes of medicine shortages in Namibia also seen in other parts of the world?

3.3 Which causes of medicine shortages in Namibia can be solved or mitigated?

3.4 Which causes of medicine shortages are difficult to solve?

# **4.** **NAMIBIAN PHARMACEUTICAL REGULATORY ENVIRONMENT**

4.1 What is NMRC’s role in the medicine supply chain?

4.2 How does pharmaceutical regulation in Namibia differ from other countries?

4.3 How does Namibia's medicines regulatory system affect pharmaceutical distribution and medicine availability?

4.4 What challenges in Namibia's pharmaceutical regulation affect medicine availability exist?

4.5 How has pharmaceutical regulation in Namibia and globally changed pre- and post- COVID-19?

*• How has this affected the medicine supply?*

# **5.** **SOLUTIONS FOR MEDICINES SHORTAGES IN NAMIBIA**

5.1 What is the role of national medicines regulatory authorities in addressing medicines shortages in Namibia?

5.2 What role of the NMRC currently play in managing medicines shortages in Namibia?

1. *What are the current gaps?*
2. *What additional measures should be put in place to address medicine shortages?*

5.3 Does the NMRC have a national tracking system for monitoring potential shortages?

1. If yes, how is it used in managing medicines shortages?

5.4 What other stakeholders should be involved in addressing medicines shortages in Namibia

*• What role should they play?*

**Ending script:**
*The interview ends here. Thank you for participating. The outcome of the study can be shared with you if required.*

---END---

**APPENDIX C: INTERVIEW GUIDE – PHARMACEUTICAL SUPPLIERS**

**PART I: CONSENT SCRIPT:**

**Introduction:** Welcome and thank you for agreeing to be interviewed. As previously mentioned, my name is xxx, and I am a fourth-year Bachelor of Pharmacy student at the University of Namibia.

Interview conduct and ethics: As communicated during the recruitment process, the interview should take between 30-40 minutes. All information obtained during the interview will be treated with strict confidentiality. Your identity will be kept anonymous, and any personal information shared during the interview will be used solely for research purposes. The interview is being recorded to help ensure complete and accurate documentation of your responses, and it will be destroyed after transcribing it and only the researcher and authorised personnel involved in this study will have access to the data.

**Risks and benefits:**

1. There are no risks involved. However, if some questions evoke emotional responses or discomfort at any point during the interview and you feel uncomfortable or distressed, you have the right to decline to answer any question or terminate the interview.
2. There are no direct benefits to you for participating in this study. However, your contribution will assist in advancing knowledge around medicine shortages in Namibia and may benefit future health service provision and research endeavours.

Voluntary participation: Your participation in this study is entirely voluntary. You have the right to refuse participation or withdraw from the study at any time without providing a reason.

Do you have any questions?

Do you give your informed consent to be interviewed? YES/NO

--------------------

**PART II: INTERVIEW**
**1. GENERAL QUESTION**
**Participant’s role and experience:**

|  | **Supplier** | **Role** | **Years of experience in procurement and sourcing** |
| --- | --- | --- | --- |
| **1.** |  |  |  |
| **2.** |  |  |  |
| **3.** |  |  |  |

# **2. MEDICINES AVAILABILITY IN NAMIBIA**

2.1 How would you describe the current state of medicines availability in Namibia?

1. *Please provide examples to illustrate this.*

2.2 How does this situation compare globally?

2.3 How does current medicine availability in Namibia compare to the past 5 years?

2.4 Which medicines or classes are frequently out of stock or in short supply in Namibia?

1. *What are the reasons for these stockouts/ shortages?*

2.5 Which medicines or classes are less affected by supply shortages?

2.6 Do medicine shortages affect the cost of medicines in Namibia?

1. *If so, how?*

2.7 How do medicine shortages affect patients in the public sector?

2.8 How do medicine shortages affect patients in the private sector?

## **3. FACTORS ASSOCIATED WITH MEDICINE SHORTAGES**

3.1 In your opinion, what are the top three causes of medicine shortages in Namibia?

1. *Are some of these most problematic?*
2. *Which ones and why?*
   1. Are the causes of medicine shortages in Namibia also seen in other parts of the world?
   2. Which causes of medicine shortages in Namibia can be solved or mitigated?
   3. Which causes of medicine shortages are difficult to solve

## **4. NAMIBIAN PHARMACEUTICAL MARKET**

4.1 What unique characteristics, if any, define the Namibian pharmaceutical market compared to other countries?

1. *How do these characteristics impact the availability of medicines in Namibia?*
2. *Are any of these characteristics particularly problematic? If so, why?*

4.2 What aspects of Namibia's medicines regulatory system if any, affect pharmaceutical distribution?

1. *How do these regulatory aspects influence medicine availability in Namibia? • Are any of these regulatory features especially problematic? If so, why?*

4.3 Has the frequency or extent of medicine shortages in Namibia changed since the COVID-19 pandemic?

1. *If yes, how?*

## **5. SOLUTIONS FOR MEDICINES SHORTAGES IN NAMIBIA**

5.1 What measures, if any, does your organisation use to address medicine shortages?

5.2 How do you prioritise customers during shortages?

5.3 Do you have a tracking system for monitoring potential shortages?

• *If so, how is it used to address shortages?*

5.4 What role do pharmaceutical suppliers currently play in addressing medicine shortages in Namibia

5.5 What measures, if any, does NMRC have in place to address medicine shortages?

5.6 What role should NMRC ideally play in addressing medicines shortages in Namibia?

5.7 Which other stakeholders (apart from pharmaceutical suppliers and NMRC) should be involved in addressing medicine shortages in Namibia, and what roles should they play?

***Ending script: The interview ends here. Thank you for participating. The outcome of the study can be shared with you if required.***

**--- END---**
